# Alisol A-24-acetate promotes glucose uptake via activation of AMPK in C2C12 myotubes

**DOI:** 10.1186/s12906-019-2802-3

**Published:** 2020-01-29

**Authors:** Jia-xiang Chen, Hai-yan Li, Tian-tian Li, Wen-cheng Fu, Xin Du, Chun-hui Liu, Wen Zhang

**Affiliations:** 1 School of Life Sciences, East China Normal University, 500 Dongchuan Road, Shanghai, 200241 China; 2 China national institute of standardization, 4 Zhichun Road, Beijing, 100191 China

**Keywords:** Alisol A-24-acetate, C2C12 myotubes, Glucose uptake, AMPK

## Abstract

**Background:**

Alisol A-24-acetate (AA-24-a) is one of the main active triterpenes isolated from the well-known medicinal plant *Alisma orientale* (Sam.) Juz., which possesses multiple biological activities, including a hypoglycemic effect. Whether AA-24-a is a hypoglycemic-active compound of *A. orientale* (Sam.) Juz. is unclear. The present study aimed to clarify the effect and potential mechanism of action of AA-24-a on glucose uptake in C2C12 myotubes.

**Method:**

Effects of AA-24-a on glucose uptake and GLUT4 translocation to the plasma membrane were evaluated. Glucose uptake was determined using a 2-(N-(7-nitrobenz-2-oxa-1,3-diazol-4-yl) amino)-2-deoxyglucose (2-NBDG) uptake assay. Cell membrane proteins were isolated and glucose transporter 4 (GLUT4) protein was detected by western blotting to examine the translocation of GLUT4 to the plasma membrane. To determine the underlying mechanism, the phosphorylation levels of proteins involved in the insulin and 5′-adenosine monophosphate-activated protein kinase (AMPK) pathways were examined using western blotting. Furthermore, specific inhibitors of key enzymes in AMPK signaling pathway were used to examine the role of these kinases in the AA-24-a-induced glucose uptake and GLUT4 translocation.

**Results:**

We found that AA-24-a significantly promoted glucose uptake and GLUT4 translocation in C2C12 myotubes. AA-24-a increased the phosphorylation of AMPK, but had no effect on the insulin-dependent pathway involving insulin receptor substrate 1 (IRS1) and protein kinase B (PKB/AKT). In addition, the phosphorylation of p38 mitogen-activated protein kinase (MAPK) and the AKT substrate of 160 kDa (AS160), two proteins that act downstream of AMPK, was upregulated. Compound C, an AMPK inhibitor, blocked AA-24-a–induced AMPK pathway activation and reversed AA-24-a–induced glucose uptake and GLUT4 translocation to the plasma membrane, indicating that AA-24-a promotes glucose metabolism via the AMPK pathway in vitro. STO-609, a calcium/calmodulin-dependent protein kinase kinase β (CaMKKβ) inhibitor, also attenuated AA-24-a–induced glucose uptake and GLUT4 translocation. Moreover, STO-609 weakened AA-24-a-induced phosphorylation of AMPK, p38 MAPK and AS160.

**Conclusions:**

These results indicate that AA-24-a isolated from *A. orientale* (Sam.) Juz. significantly enhances glucose uptake via the CaMKKβ-AMPK-p38 MAPK/AS160 pathway.

## Background

Type 2 diabetes mellitus (T2DM) has become one of the most serious global health issues and is mainly attributed to excess body weight and inactivity. According to the International Diabetes Federation, 425 million people worldwide are currently suffering from T2DM [1]. T2DM is characterized by over-nutrition and insulin resistance, which lead to high blood glucose levels and vascular complications that include cardiovascular diseases, diabetic nephropathy and diabetic retinopathy [2–4]. Lowering blood glucose levels reduces the incidence of such complications. Thus it is pivotal to T2DM research that we find ways to promote glucose uptake in skeletal muscle and lower glucose production in the liver.

Glucose uptake in skeletal muscle is regulated by two distinct pathways: 1) stimulation by insulin through IRS1 and phosphatidylinositol 3 (PI3)-kinase [5]; and 2) stimulation by muscle contraction and exercise through the activation of AMPK [6]. AMPK has long been regarded as a promising therapeutic target for metabolic syndrome. AMPK belongs to a family of serine/threonine kinases, acting as a cellular energy sensor that monitors the AMP: ATP ratio to maintain cellular homeostasis [7]. The Thr^172^ site on the α subunit of AMPK is crucial to AMPK regulation [8]. In its activated state, AMPK can phosphorylate multiple kinases and other downstream target proteins to exert its various functions. AMPK can phosphorylate and inhibit acetyl-CoA carboxylase (ACC), promoting fatty acid transportation and β-oxidation [9]. AMPK also phosphorylates and inhibits the AS160, which ultimately promotes the translocation of GLUT4 [6]. Translocation of GLUT4 from vesicles to the plasma membrane is a critical step in cellular glucose uptake. Furthermore, studies have revealed that AMPK can also phosphorylate and activate p38 MAPK [10], whose role in promoting cell glucose uptake has already been clarified [10, 11]. AMPK is regulated by various upstream kinases, including but not limited to CaMKKβ, transforming growth factor (TGF) β-activated kinase 1 (TAK1) and liver kinase B1 (LKB1) [12].

*Alisma orientale* (Sam.) Juz., a well-known medicinal plant, is mainly found in China, Russia, Japan, Mongolia and North India. Its dried rhizome, Rhizoma Alismatis, is a well-known traditional Chinese medicine that has been widely used in China for more than 1000 years. Pharmacological research has revealed that it has multiple biological activities that include diuretic, anti-inflammatory, anti-tumor, hepatoprotective, hypolipidemic and hypoglycemic effects [13–18].

Alisol A-24-acetate (AA-24-a) is one of the main active triterpenes that have been isolated from Rhizoma Alismatis. While it has been reported that AA-24-a can lower cholesterol [19] and prevent hepatic steatosis [20], its potential effect on glucose metabolism has not been investigated. Glucose uptake by peripheral tissues such as skeletal muscles and adipocytes is important for the maintenance of glucose homeostasis [21], and is one mechanism for prevention or amelioration of hyperglycemia and T2DM. Because the skeletal muscles are responsible for approximately 75% of glucose uptake, we chose to use myotubes from a murine cell line, C2C12, to evaluate the effect of AA-24-a on glucose metabolism. While our preliminary study revealed that AA-24-a significantly promoted glucose consumption in C2C12 myotubes (unpublished results), not much is known about its effect on glucose uptake in myotubes. We hypothesized that triterpenes AA-24-a isolated from Rhizoma Alismatis might improve glucose metabolism by promoting glucose uptake via the IRS1/PI3-kinase pathway or the AMPK pathway. To test this hypothesis, we examined the expression of key components of the IRS1/PI3-kinase and AMPK pathways. And then, specific kinase inhibitors were used to investigate the mechanism of AA-24-a on glucose uptake in C2C12 myotubes.

## Methods

### Chemicals and reagents

We purchased AA-24-a extracted from Rhizoma Alismatis from Chengdu Herbpurify (purity 98.81% by HPLC). Cell Counting Kit (CCK), secondary antibodies and insulin were obtained from Yeasen Biotech. Dimethyl sulfoxide (DMSO) and 5-aminoimidazole-4-carboxamide ribonucleotide (AICAR) were purchased from Sigma. Compound C and STO-609 were purchased from Sellbeck chemicals. Mem-PER Plus Membrane Protein Extraction Kit and 2-(N-[7-nitrobenz-2-oxa-1,3-diazol-4-yl] Amino)-2-deoxyglucose (2-NBDG) were purchased from Thermo. Foetal bovine serum (FBS) was purchased from BOVOGEN, and other cell culture materials, including Dulbecco’s modified Eagle’s medium (DMEM), horse serum, antibiotic/antimycotic and trypsin solutions were obtained from GIBCO Life Technologies. We used antibodies against the following proteins: AMPKα, phospho-AMPKα (Thr172), acetyl-CoA carboxylase (ACC), phospho-ACC (Ser79), AS160, phospho-AS160 (Ser588), phospho-p38 MAPK (Thr180/Tyr182), IRS-1, phospho-AKT (Ser473) and phospho-AKT (Thr308), which were purchased from Cell Signaling Technology; GLUT4 and phosphor-IRS1 (Tyr632), which were purchased from Abcam; and AKT, ATP1A1, p38 MAPK and β-actin, which were obtained from Proteintech.

### Cell culture and differentiation

The C2C12 mouse myoblasts were obtained from The National Center for Drug Screening (Shanghai, China). C2C12 mouse myoblasts were maintained in DMEM supplemented with 10% (v/v) FBS, streptomycin (100 U/mL), and penicillin (100 U/mL) (37 °C; 5% CO_2_). Cells were seeded into cell culture plates at a density of 5 × 10^4^ cells/mL. After 24 h (about 70% confluence), the medium was switched to DMEM supplemented with 2% (v/v) horse serum and replaced after 2, 4 and 6 days of culture. Experiments were initiated on day 7, when myotube differentiation was complete. Cells were serum-starved for 6 h before being subjected to any experimental treatment.

### CCK-8 assay

Fully differentiated C2C12 mouse myotubes were cultured in 96-well plates and treated with AA-24-a at varying concentrations and a range of time periods. CCK-8 reagents (included in Cell Counting Kit, 10 μL) were added to each well 1 h before harvesting. Cells were maintained in an incubator (37 °C; 5% CO_2_) for 1 h, then absorbance was measured at 450 nm. Cell viability was calculated by the following formula:

$$ \mathrm{Cell}\ \mathrm{Viability}\%=\frac{{\mathrm{OD}}_{\mathrm{Sample}}-{\mathrm{OD}}_{\mathrm{Blank}}}{{\mathrm{OD}}_{\mathrm{Control}}-{\mathrm{OD}}_{\mathrm{Blank}}}\mathrm{x}100\% $$

### 2-NBDG uptake

Cell glucose uptake was determined as a measure of 2-NBDG uptake using the following procedure. Fully differentiated C2C12 mouse myotubes were cultured in black 96-well plates and treated with AA-24-a at varying concentrations and a range of time periods. At 1 h before harvest, cells were washed twice with warm sterile phosphate buffered saline (PBS) then the medium was switched to glucose-free DMEM supplemented with 0.2% fatty-acid-free bovine serum albumin (BSA). After 1 h, cells were washed once with sterile PBS (warmed up at 37 °C) and then incubated with the same medium containing 80 μM 2-NBDG for 30 min. Cells were then washed once more with warm sterile PBS before measuring the fluorescence intensity of each well (Ex485nm, Em520nm). Cell glucose uptake was calculated by following formula:

$$ \mathrm{Cell}\ \mathrm{glucose}\ \mathrm{uptake}=\frac{{\mathrm{FI}}_{\mathrm{Sample}}-{\mathrm{FI}}_{\mathrm{Blank}}}{{\mathrm{FI}}_{\mathrm{Control}}-{\mathrm{FI}}_{\mathrm{Blank}}} $$

### Western blotting

After treatment cells were washed twice with ice-cold PBS then harvested in radioimmunoprecipitation assay lysis buffer (150 mM sodium chloride, 1.0% Triton X-100, 0.5% sodium deoxycholate, 0.1% sodium dodecyl sulfate [SDS] and 50 mmol/L Tris; pH 8.0) containing protease and phosphatase inhibitors. Cell membrane proteins were extracted using a Mem-PERa Plus Membrane Protein Extraction Kit according to the manufacturer’s protocol. The protein concentrations were determined using a bicinchoninic acid (BCA) Protein Assay Kit (Yeasan) according to the manufacturer’s protocol. Equal quantities of protein were separated on 10% SDS-polyacrylamide gel electrophoresis (SDS-PAGE) gels and transferred to nitrocellulose membranes. The membranes were blocked in 5% non-fat drymilk solution at room temperature for 1 h and incubated overnight at 4 °C with primary antibodies. After three washes in tris-buffered saline with 0.1% Tween 20, the membranes were incubated for 1 h with secondary antibodies at room temperature. The blots were washed and then visualized on an Odyssey CLx Imaging System. All blots were analyzed by Image-Pro Plus Software.

### Statistical analysis

Results are expressed as mean ± standard deviation (SD). Statistical significance among different experimental groups was determined by one-way analysis of variance followed by Duncan’s multiple-comparisons test using SPSS software (IBM; Armonk, NY, USA). Differences between two groups were identified using Student’s t-test. *P* values of < 0.05 and < 0.01 were considered to be statistically significant and extremely significant, respectively.

## Results

### AA-24-a promotes glucose uptake and GLUT4 translocation in C2C12 myotubes

**Fig. 1:**
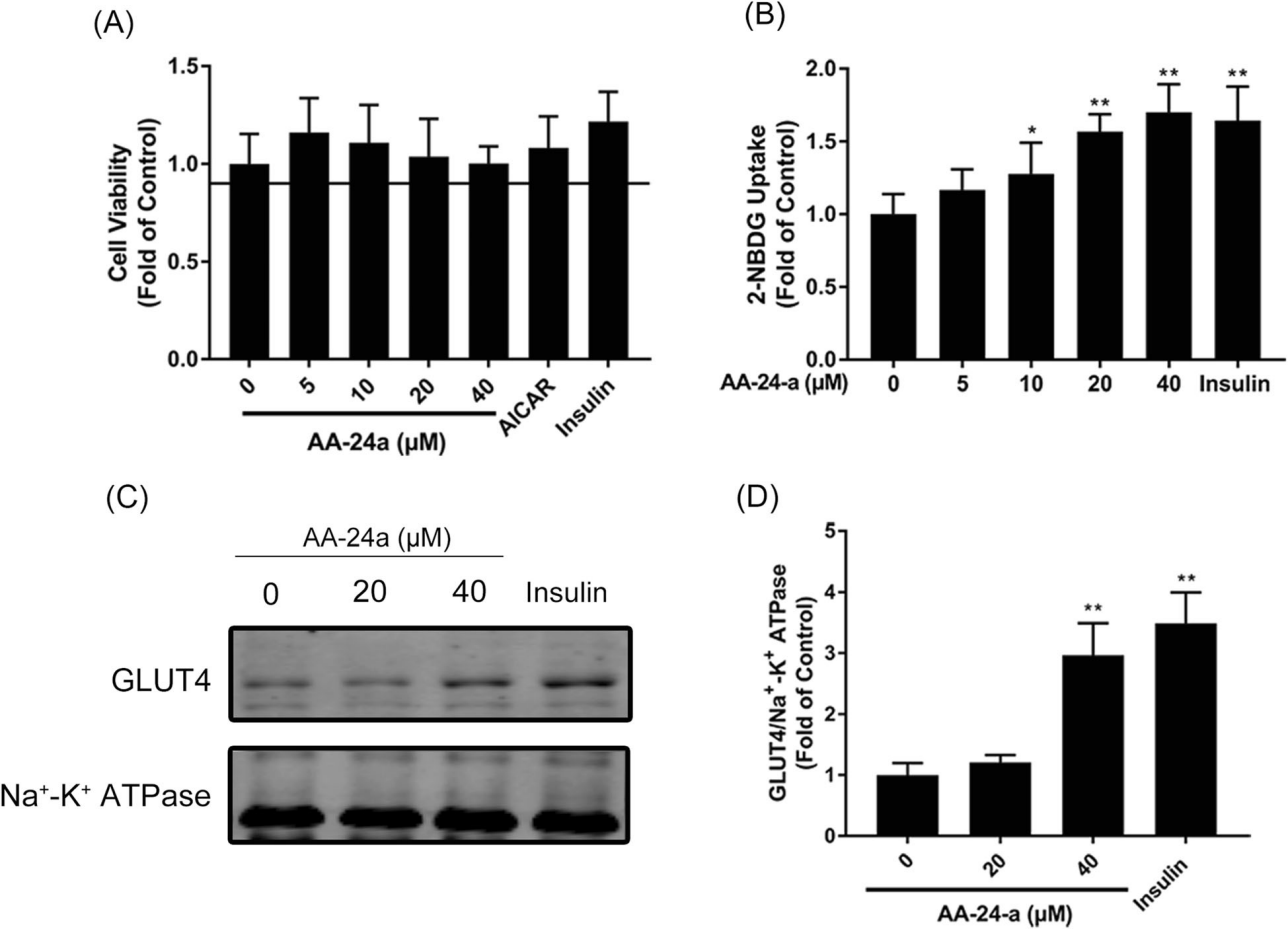
Effects of AA-24-a on GLUT4 translocation and glucose uptake in C2C12 myotubes. Cells were treated with AA-24-a for 12 h or insulin (100 nM) for 15 min. **a** CCK-8 assay of cell viability. **b** 2-NBDG assay of glucose uptake. Western blotting (**c**) and quantification (**d**) of GLUT4 in cell membrane proteins. Values are expressed as means ± SD (*n* = 3). ^*^P < 0.05, ^**^ *P* < 0.01, vs. control group

To determine the effects of AA-24-a treatment on the viability of C2C12 myotubes, we performed a CCK-8 assay. AA-24-a had no effect on cell viability in cultures of differentiated C2C12 myotubes treated with 0–40 μM AA-24-a for 12 h (Fig. 1a, *P* > 0.05) and 24 h (Additional file 1: Figure S1).

Next we investigated the effects of AA-24-a on glucose uptake and GLUT4 translocation. Differentiated C2C12 myotubes were treated with 0–40 μM AA-24-a for 12 h or insulin (100 nM, 15 min) as a positive control. As shown in Fig. 1b, treatment with 10, 20 and 40 μM AA-24-a significantly promoted cell glucose uptake by factors of 1.28-, 1.57- and 1.70-fold, respectively, compared with the control group (*P* < 0.01 or 0.05). After treatment cells with 40 μM AA-24-a for 12 h, the level of GLUT4 protein in the plasma membrane was significantly increased by 2.97-fold compared with those of control cells (Fig. 1c, d, *P* < 0.01). These results indicated that AA-24-a promoted GLUT4 translocation and cell glucose uptake in C2C12 myotubes.

### AA-24-a promotes glucose uptake and GLUT4 translocation via AMPK pathway in C2C12 myotubes

**Fig. 2:**
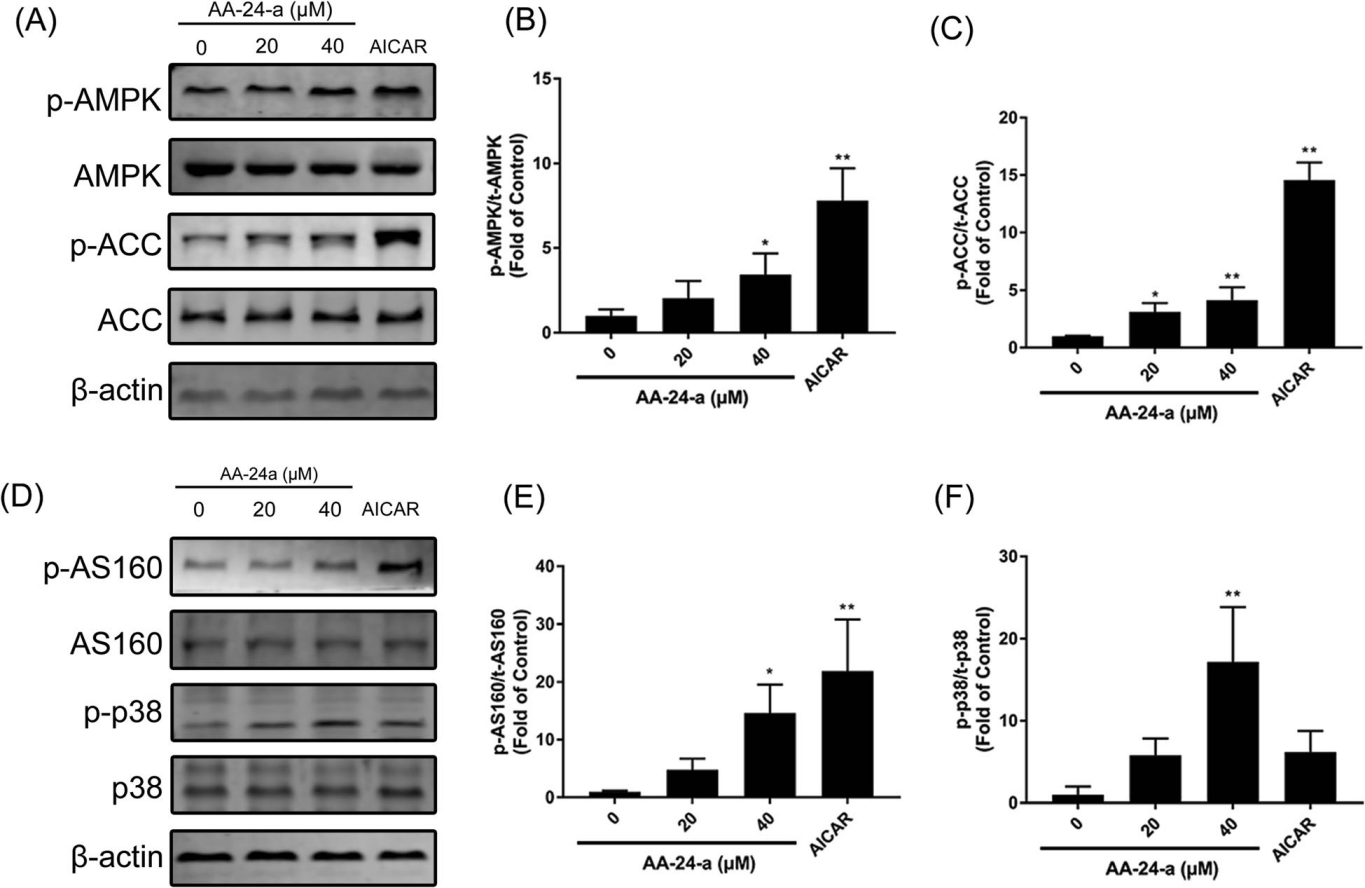
Effects of AA-24-a on phosphorylation of AMPK, ACC, AS160 and p38 MAPK. C2C12 myotubes were treated with AA-24-a for 12 h or AICAR (500 μM) for 1 h. Western blotting (**a**) and quantification (**b, c**) of phospho-AMPK and phospho- ACC in whole cell lysates. Western blotting (**d**) and quantification (**e, f**) of phospho-AS160 and phospho- p38 MAPK. Values are expressed as means ± SD (*n* = 3 or 4). ^*^ *P* < 0.05, ^**^ *P* < 0.01, vs. control group

To explore the underlying molecular mechanism by which AA-24-a promotes cell glucose uptake, the phosphorylation levels of proteins involved in the insulin and AMPK pathways were examined. As shown in Additional file 2: Figure S2, AA-24-a had no effect on the phosphorylation levels of IRS1 (Tyr632) (Additional file 2: Figure S2A**)** or AKT (Ser473 and Thr308) (Additional file 2: Figure S2A and 2B). However, AA-24-a upregulated the phosphorylation levels of AMPK (Thr172) and its downstream proteins ACC (Ser79) and AS160 (Ser588) in a time-dependent manner (Additional file 3: Figure S3). Treatment C2C12 myotubes with AA-24-a (40 μM) for 12 h significantly upregulated the levels of phosphorylated AMPK, ACC (Figs. 2a-c), p38 and AS160 (Figs. 2d-f) by 3.44-, 4.13-, 17.20- and 14.63-fold, respectively, compared with the control group (Fig. 2, *P* **<** 0.05 or 0.01). These results indicated that AA-24-a activated the AMPK pathway.

**Fig. 3:**
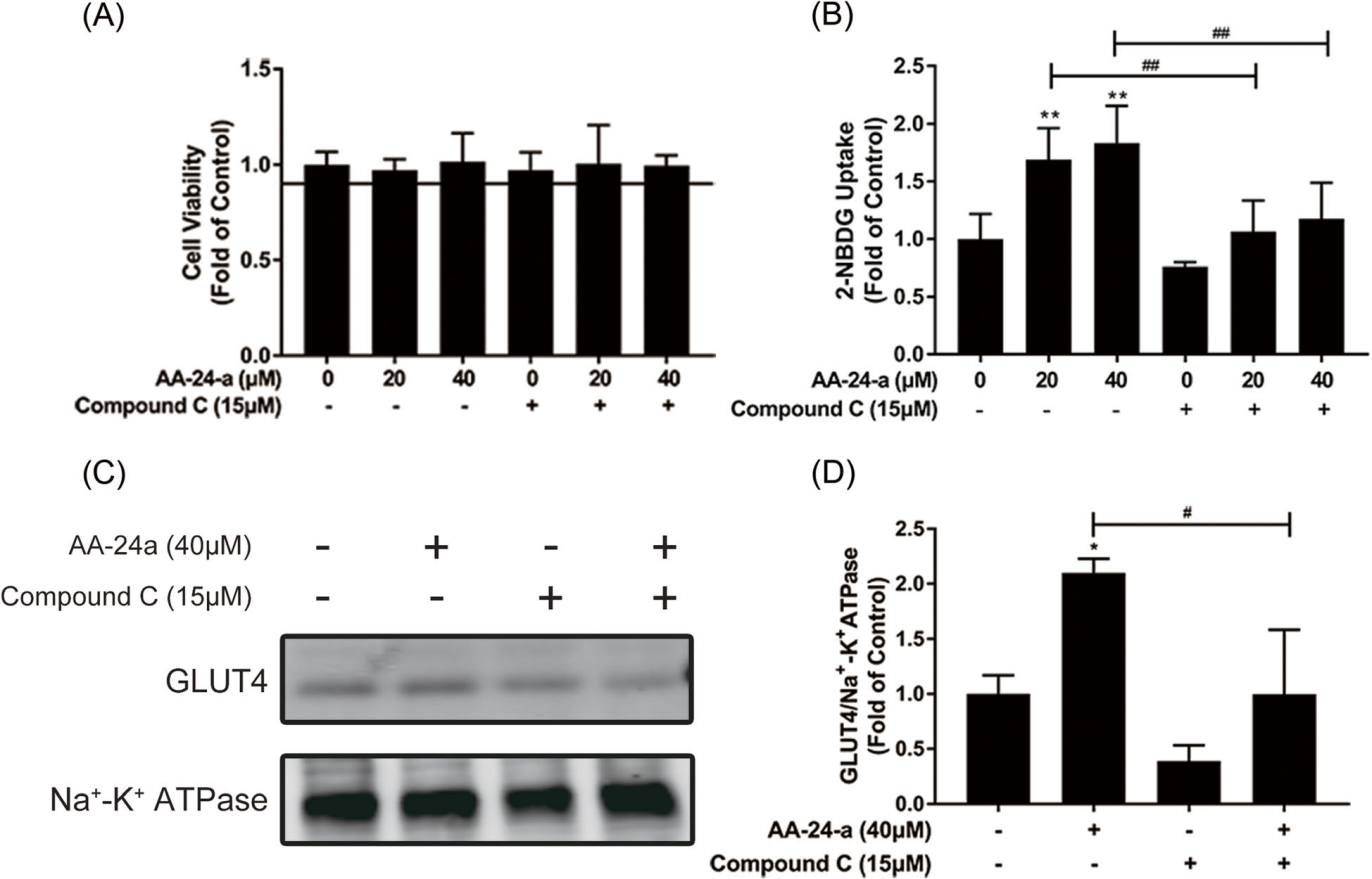
Effects of AMPK inhibitor compound C on AA-24-a-stimulated glucose uptake and GLUT4 translocation. C2C12 myotubes were incubated in the presence or absence of compound C (15 μM) for 1 h followed by exposure to AA-24-a for 12 h. (**a**) CCK-8 assay of cell viability. **b** 2-NBDG assay of glucose uptake. Western blotting (**c**) and quantification (**d**) of GLUT4 in cell membrane proteins. Values are expressed as means ± SD (n = 3). ^*^ *P* < 0.05, ^**^ *P* < 0.01, vs. control group; ^#^ *P* < 0.05, ^##^ *P* < 0.01, vs. AA-24-a group

**Fig. 4:**
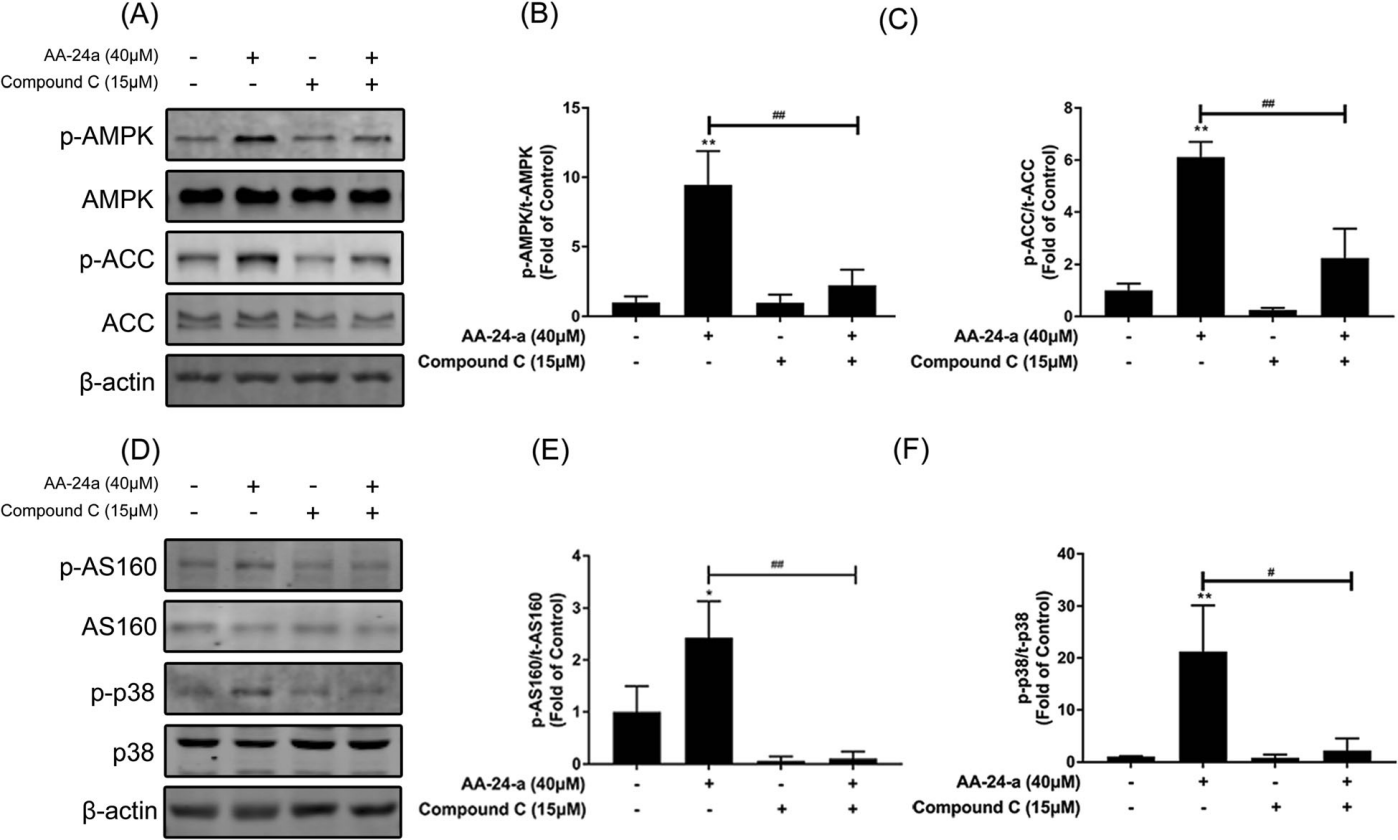
Effects of AMPK inhibitor compound C on AA-24-a-induced phosphorylation of AMPK, ACC, AS160 and p38 MAPK. Cells were incubated in the presence or absence of compound C (15 μM) for 1 h followed by exposure to AA-24-a (40 μM) for 6 h. Western blotting of whole cell lysates to detect phosphorylation of AMPK, ACC, AS160 and p38 MAPK. Western blotting (**a**) and quantification (**b**, **c**) of phospho-AMPK and phospho-ACC. Western blotting (**d**) and quantification (**e**, **f**) of phospho-AS160 and phospho-p38 MAPK. Values are expressed as means ± SD (*n* = 3 or 4). * *P* < 0.05, ** *P* < 0.01, vs. control group; # *P* < 0.05, ## *P* < 0.01, vs. AA-24-a group

To confirm whether the promotional effect of AA-24-a on glucose uptake and GLUT4 translocation was mediated through AMPK activation, we pretreated the myotubes with compound C (15 μM), an AMPK-specific inhibitor, for 1 h prior to AA-24-a (40 μM) treatment for 12 h (in the presence of compound C). As shown in Fig. 3, AA-24-a–stimulated glucose uptake (Fig. 3b) and GLUT4 translocation (Fig. 3c, d) decreased in myotubes pretreated with compound C (*P* < 0.05 or 0.01). Furthermore, compound C blocked the upregulation of phosphorylation of AMPK, ACC (Figs. 4a-c), AS160 and p38 (Figs. 4d-f) induced by AA-24-a treatment (*P* < 0.05 or 0.01 vs. AA-24-a treatment alone). These results indicated that AA-24-a stimulated glucose uptake in C2C12 myotubes via the AMPK pathway.

### AA-24-a activates AMPK pathway via CaMKKβ in C2C12 myotubes

**Fig. 5:**
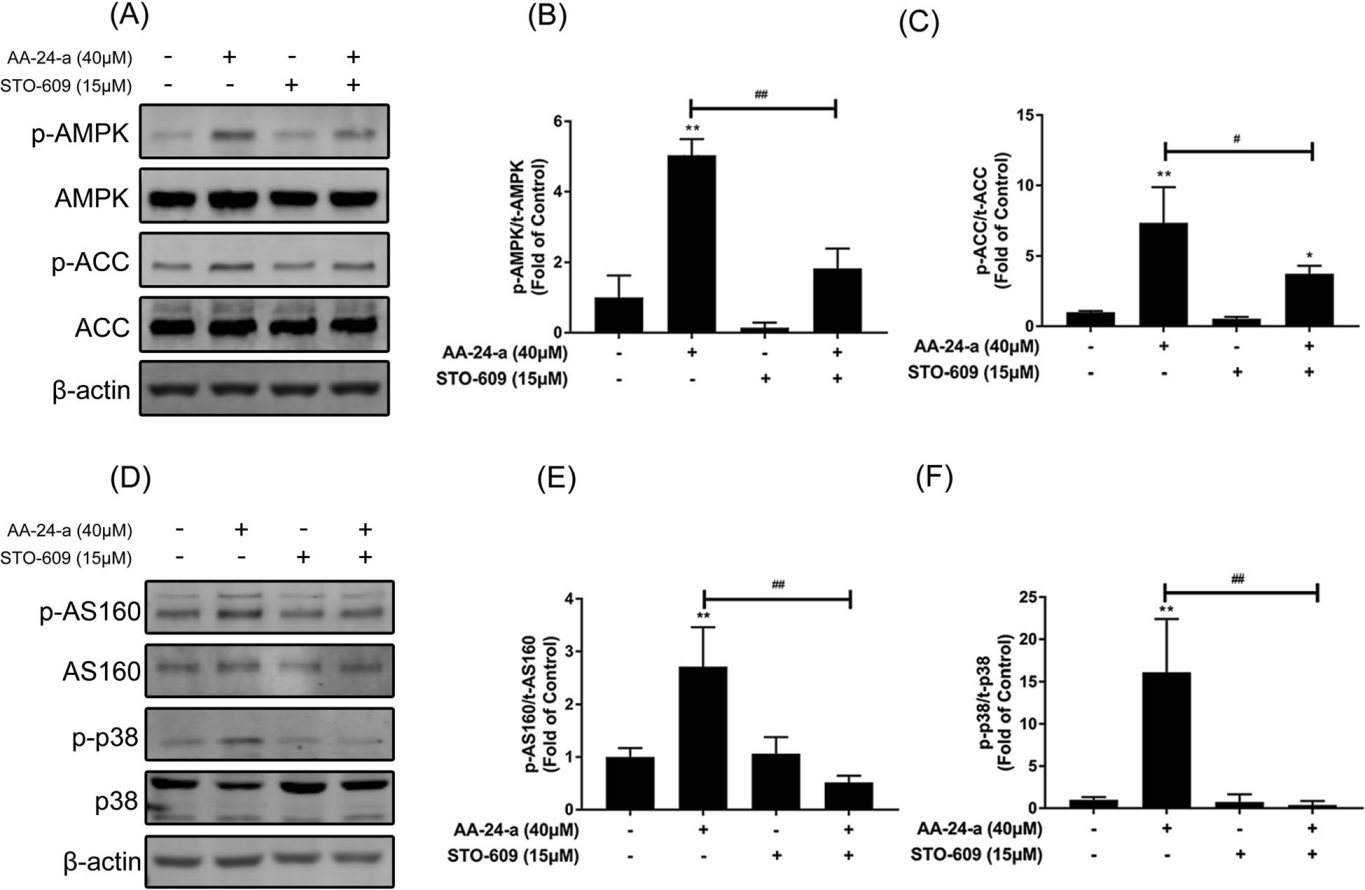
Effect of CaMKKβ inhibitor STO-609 on AA-24-a-induced phosphorylation of AMPK, ACC, AS160 and p38 MAPK. Myotubes were incubated in the presence or absence of STO-609 (15 μM) for 1 h followed by exposure to AA-24-a (40 μM) for 6 h. Western blotting of whole cell lysates to detect phosphorylation of AMPK, ACC, AS160 and p38 MAPK. Western blotting (**a**) and quantification (**b**, **c**) of phospho-AMPK and phospho-ACC. Western blotting (**d**) and quantification (**e**, **f**) of phospho-AS160 and phospho-p38 MAPK. Values are expressed as means ± SD (*n* = 3 or 4). ^*^ *P* < 0.05, ^**^ *P* < 0.01, vs. control group; ^#^ *P* < 0.05, ^##^ *P* < 0.01, vs. AA-24-a group

**Fig. 6:**
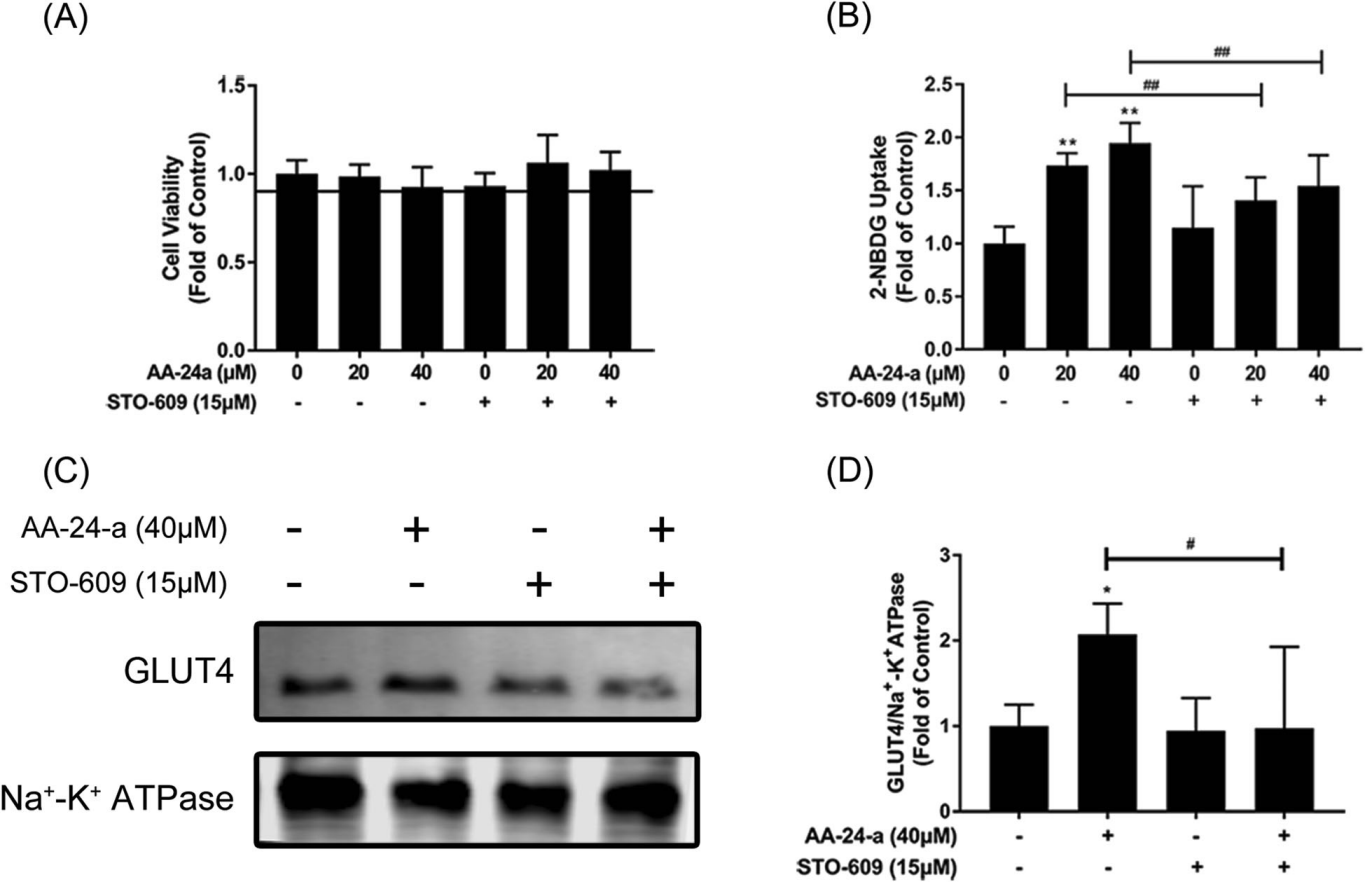
Effects of CaMKKβ inhibitor STO-609 on AA-24-a-stimulated glucose uptake and GLUT4 translocation. C2C12 myotubes were incubated in the presence of STO-609 (15 μM) for 1 h followed by exposure to AA-24-a for 12 h. **a** CCK-8 assay of cell viability. **b** 2-NBDG assay of glucose uptake. Western blotting (**c**) and quantification (**d**) of GLUT4 in cell membrane proteins. Values are expressed as means ± SD (*n* = 3). ^*^ *P* < 0.05, ^**^ *P* < 0.01, vs. control group; ^#^ *P* < 0.05, ^##^ *P* < 0.01, vs. AA-24-a group

Multiple kinases can activate AMPK, including CaMKKβ and LKB1 [12]. It has also been reported that CaMKKβ acts as an upstream effector of AMPKa2 in the activation of glucose uptake [22]. To investigate the possible role of CaMKKβ in AA-24-a–mediated glucose uptake, a CaMKKβ inhibitor (STO-609) was employed. As results shown in Fig. 5, STO-609 blocked AA-24-a–induced phosphorylation of AMPK and its downstream proteins ACC (Figs. 5a-c), p38 and AS160 (Figs. 5d-f; *P* < 0.05 or 0.01 vs. AA-24-a treatment alone), indicating that AA-24-a activated the AMPK pathway via CaMKKβ. Furthermore, pretreatment with STO-609 blocked AA-24-a–induced glucose uptake (Fig. 6b; *P* < 0.01 vs. AA-24-a treatment alone) and GLUT4 translocation (Fig. 6C, D; *P* < 0.05 vs. AA-24-a treatment alone). These results indicated that AA-24-a promotes glucose uptake through the CaMKKβ-AMPK-p38 MAPK/AS160 pathway.

## Discussion

Because muscles play a key role in the regulation of energy balance and are considered the most important tissue for glucose disposal [23], we used C2C12 myotubes in this pilot study demonstrating the stimulation of glucose uptake by AA-24-a from Rhizoma Alismatis.

GLUT4 translocation is central to glucose metabolism. The translocation of GLUT4 from intracellular vesicles to the plasma membrane is the most important step in regulating glucose uptake, and can be promoted by insulin as well as AMPK [24]. By extracting membrane proteins from cell lysates, we found that treatment of AA-24-a led to significantly increased GLUT4 levels in the plasma membrane, which indicating that AA-24-a enhanced GLUT4 translocation. This result was in agreement with the upregulation of glucose uptake induced by AA-24-a treatment in C2C12 myotubes.

As mentioned above, skeletal muscle glucose uptake is regulated by two distinct pathways: the insulin-dependent IRS1/PI3K pathway [5] and the AMPK pathway, which is activated by muscle contraction or exercise [6]. We found that AA-24-a did not have effects on IRS1 or AKT but strongly activated AMPK, suggesting that AMPK pathway probably involved in AA-24-a stimulates glucose uptake.

AMPK plays important roles in maintaining cell energy and glucose homeostasis [7]. Once activated, it accelerates ATP-generating catabolic pathways, including those for lipid metabolism, glucose uptake and fatty acid oxidation, by directly regulating the key metabolic enzymes [9, 12]. Because of its involvement in the metabolic syndrome, AMPK has been extensively studied. Metformin and AICAR are powerful AMPK agonists, with the former widely used in T2DM treatment [25]. In this study, we observed that AA-24-a increased the phosphorylation of AMPK on Thr172, which is the most important regulatory site of AMPK. AA-24-a also increased the phosphorylation of ACC, a downstream target of AMPK that is commonly used as proof of AMPK activation. Moreover, AMPK inhibitor compound C attenuated AA-24-a–induced AMPK activation, glucose uptake and GLUT4 translocation. Thus, our data indicate that AA-24-a increases cell glucose uptake and GLUT4 translocation via the AMPK pathway. A previous study that demonstrated significant effects of AA-24-a on the AMPK pathway in terms of amelioration of hepatic steatosis and inhibition of inflammation in HepG2 cells [20] accords with our results. Another study in C57BL/6 mice and WRL-68 liver cells also indicated that AA-24-a inhibits oxidative stress and stimulates autophagy by activating AMPK [26].

AS160 is an important downstream protein of the insulin pathway [5, 6]. Recent research efforts have revealed that AMPK phosphorylates AS160 to inactivate it, eventually leading to upregulation of glucose uptake and GLUT4 translocation [6]. Our study demonstrated that the AA-24-a–mediated phosphorylation of AS160 was blocked by AMPK inhibitor compound C, indicating that AA-24-a–mediated inactivation of AS160 by AMPK is one of the underlying mechanisms of upregulation of glucose uptake and GLUT4 translocation.

Recent studies have provided evidence that p38 MAPK plays a pivotal role in glucose uptake in skeletal muscles [10, 11]. Many studies have investigated the connection between AMPK and p38 MAPK. Activation of p38 MAPK is almost completely abolished in various cells expressing the dominant-negative AMPK mutant [27, 28]. Therefore, it seems clear that p38 MAPK is a downstream protein of AMPK that participates in AMPK-dependent regulation of glucose uptake. In the current study, we found that AA-24-a–induced p38 MAPK activation was completely blocked by the AMPK inhibitor compound C, which suggests that AA-24-a–induced glucose uptake occurs via the AMPK-p38 MAPK pathway in C2C12 myotubes.

AMPK is regulated by LKB1, CaMKKβ and TAK1 among other upstream kinases [12]. We found that preincubation of STO-609, an established CaMKKβ inhibitor, reversed the AA-24-a–induced upregulation of glucose uptake and GLUT4 translocation, as well as activation of the AMPK pathway, demonstrating that AA-24-a promotes glucose uptake and activates AMPK through CaMKKβ. However, Ca^2+^ ionophores, which increase cellular Ca^2+^ levels to activate CaMKKβ, also activate AMPK phosphorylation in the same cell line. Whether AA-24-a exerts its effect by regulating the Ca^2+^ ionophore concentration in cells remains to be discovered.

The above results demonstrated that AA-24-a, one of the main active triterpenes of Rhizoma Alismatis, significantly enhances glucose uptake via the CaMKKβ-AMPK-p38 MAPK/AS160 pathway in C2C12 myotubes. Then, this effect need be validated in vivo, the following studies will attempt to investigate whether AA-24-a decrease blood glucose through activation of AMPK in diabetic animal models.

## Conclusions

In summary, this pilot study has shown that AA-24-a promoted glucose uptake and GLUT4 translocation in C2C12 myotubes through a mechanism involving the CaMKKβ-mediated phosphorylation of AMPK and its downstream proteins p38 MAPK and AS160. These findings provide insight into the hypoglycemic functions of AA-24-a and raise the possibility that AA-24-a could be developed as an anti-diabetic agent.

## Supplementary information

**Additional file 1: Figure S1.** Effects of AA-24-a on cell viability in C2C12 myotubes treated with AA-24-a for 24 h.

**Additional file 2: Figure S2.** Effects of AA-24-a on proteins involved in the insulin signaling pathway as shown by western blotting of whole cell lysates from C2C12 myotubes incubated with AA-24-a (40 μM) for the indicated times. Phosphorylation (p) of two proteins were examined: IRS1 and AKT. β-actin was used as a standard.

**Additional file 3: Figure S3.** Effects of AA-24-a on proteins involved in the AMPK pathway as shown by western blotting of whole cell lysates from C2C12 myotubes incubated with AA-24-a (40 μM) for the indicated times. Phosphorylation of AMPK, ACC and AS160 were examined.

## Data Availability

The data was included in figures of the manuscript, and the raw data for this study are available upon reasonable request to the corresponding author.

## Abbreviations

2-NBDG: 2- (N- (7-Nitrobenz-2-oxa-1,3-diazol-4-yl) Amino) -2-deoxyglucose
AA-24-a: Alisol A 24-acetate
ACC: Acetyl-CoA carboxylase
AICAR: 5-aminoimidazole-4-carboxamide ribonucleotide
AMPK: 5′-adenosine monophosphate-activated protein kinase
AS160: AKT substrate of 160 kDa
BSA: Bovine serum albumin
CaMKKβ: Calcium/calmodulin-dependent protein kinase kinase β
DMEM: Dulbecco’s modified Eagle medium
FBS: Fetal bovine serum
GLUT4: Glucose transport 4
IDF: International Diabetes Federation
IRS1: Insulin receptor substrate 1
LKB1: Liver kinase B1
p38 MAPK: p38 mitogen-activated protein kinase
PI3K: Phosphoinositide 3-kinase
PKB/AKT: Protein kinase B
T2DM: Type 2 diabetes mellitus
TAK1: TGFβ-activated kinase 1
