# Supplementary figures and images for: Alisol A-24-acetate promotes glucose uptake via activation of AMPK in C2C12 myotubes
Source: BMC Complement Med Ther. 2020 Jan 29;20:22. doi: 10.1186/s12906-019-2802-3 (PMC7076831; doi:10.1186/s12906-019-2802-3)

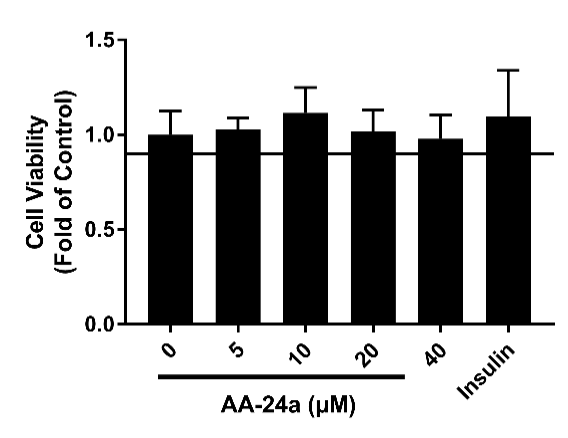

Supplement: Supplementary file 1 — Additional file 1: Figure S1. Effects of AA-24-a on cell viability in C2C12 myotubes treated with AA-24-a for 24 h. [file 12906_2019_2802_MOESM1_ESM.tif]
